# Differential Effects of Awake Glioma Surgery in “Critical” Language Areas on Cognition: 4 Case Studies

**DOI:** 10.1155/2017/6038641

**Published:** 2017-06-22

**Authors:** Djaina Satoer, Elke De Witte, Marion Smits, Roelien Bastiaanse, Arnaud Vincent, Peter Mariën, Evy Visch-Brink

**Affiliations:** ^1^Department of Neurosurgery, Erasmus MC University Medical Center, Rotterdam, Netherlands; ^2^Department of Clinical and Experimental Neurolinguistics, Free University of Brussels, Brussels, Belgium; ^3^Department of Radiology, Erasmus MC University Medical Center, Rotterdam, Netherlands; ^4^Center for Language and Cognition Groningen (CLCG), University of Groningen, Groningen, Netherlands; ^5^Department of Neurology and Memory Clinic, ZNA Middelheim, Antwerp, Belgium; ^6^Department of Neurology, Erasmus MC University Medical Center, Rotterdam, Netherlands

## Abstract

Awake surgery with electrocorticosubcortical stimulation is the golden standard treatment for gliomas in eloquent areas. Preoperatively, mostly mild cognitive disturbances are observed with postoperative deterioration. We describe pre- and postoperative profiles of 4 patients (P1–P4) with gliomas in “critical” language areas (“Broca,” “Wernicke,” and the arcuate fasciculus) undergoing awake surgery to get insight into the underlying mechanism of neuroplasticity. Neuropsychological examination was carried out preoperatively (at T1) and postoperatively (at T2, T3). At T1, cognition of P1 was intact and remained stable. P2 had impairments in all cognitive domains at T1 with further deterioration at T2 and T3. At T1, P3 had impairments in memory and executive functions followed by stable recovery. P4 was intact at T1, followed by a decline in a language test at T2 and recovery at T3. Intraoperatively, in all patients language positive sites were identified. Patients with gliomas in “critical” language areas do not necessarily present cognitive disturbances. Surgery can either improve or deteriorate (existing) cognitive impairments. Several factors may underlie the plastic potential of the brain, for example, corticosubcortical networks and tumor histopathology. Our findings illustrate the complexity of the underlying mechanism of neural plasticity and provide further support for a “hodotopical” viewpoint.

## 1. Introduction

Awake surgery is considered the golden standard treatment for low-grade gliomas (LGG) in eloquent regions to optimize tumor resection while preserving neurological and cognitive functions and hence quality of life [1, 2]. However, deficits in cognitive functions, that is, language, memory, attentional, and executive functions, occur in the (pre- and) postoperative phase of awake glioma surgery [3–5].

Eloquent regions typically include the left dominant perisylvian brain regions. DES has provided evidence for a “hodotopical” (i.e., dynamic) view of the organization of brain functions as opposed to a “topological” viewpoint (i.e., static organization of brain functions) [6–8]. Language functions are “classically” represented in cortical areas such as Broca's and Wernicke's area and in the subcortical tracts that connect different eloquent cortical regions. LGGs typically invade functional subcortical white matter tracts. However, due to the relative slow growth rate (i.e., 4 mm a year) of LGG, neural plasticity can be facilitated [9, 10]. This may be the reason that, instead of moderate to severe language problems, typically mild language disorders are observed in this patient group [11]. Despite intense intraoperative monitoring, brain tumor surgery resection may induce or aggravate the existing cognitive deficits. For a long time, complete recovery within 3 months was claimed to take place, but Satoer et al. [5]. found that cognitive recovery can continue until up to at least 1 year postoperatively. A recent review of cognition in glioma patients showed various pre- and postoperative cognitive profiles with deficits in various domains at different time-moments [12]. These findings point towards differential postoperative recovery courses of cognitive functions. Apart from individual variability in functional organization and language lateralization, other factors accounting for the potential of neuroplasticity are under debate. Tumor related characteristics (e.g., tumor volume, grade) may interfere with the course of cognitive recovery [13, 14]. Anticonvulsants and adjuvant therapy (radio- and chemotherapy) as well as the degree of seizures (frequency) may have impact on the functional cerebral network in brain tumor patients [15]. In this article, we describe 4 patients with a brain tumor in dominant perisylvian language areas in proximity of the arcuate fasciculus with differential pre- and postoperative cognitive profiles illustrating the diversity of neural plasticity processes.

## 2. Materials and Methods

### 2.1. Case Reports

**Figure 1:**
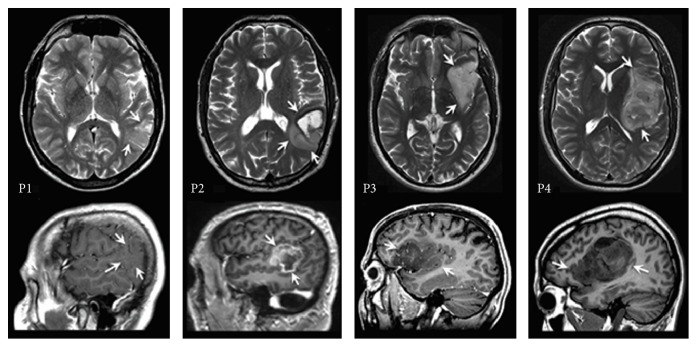
Preoperative MRI scans axial T2 weighted and sagittal T1 weighted (contrast-enhanced in P1 and in P3) sections depicting tumor localization (arrows).

**Figure 2:**
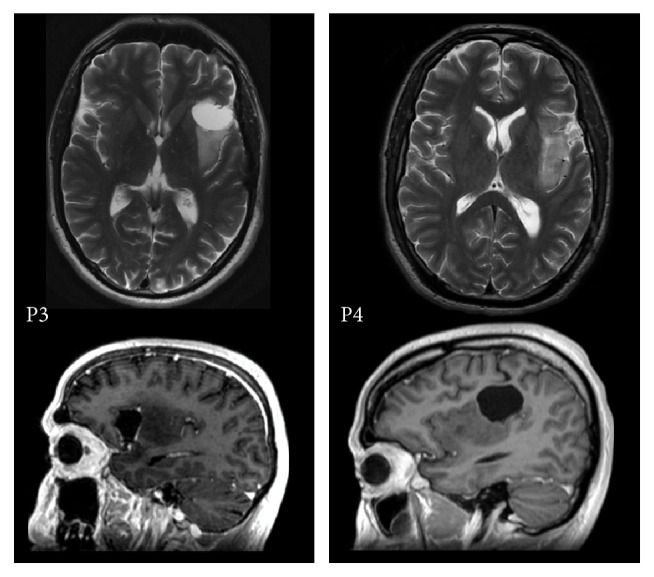
Postoperative MRI scans axial T2 weighted and sagittal T1 weighted P3 and P4.

This is a follow-up study of 4 patients that we selected based on tumor localization in perisylvian language areas. The patients (P1, P2, P3, and P4) were diagnosed with a glioma in the language dominant left hemisphere as identified with fMRI (see structural MRI scans for tumor localization in Figure 1 and resection cavity in Figure 2). The demographic and clinical characteristics of the patients are shown in Table 1. The tumor was in proximity to the posterior temporoparietal language regions “Wernicke” in patients P1, P2, and P4. In P4, the tumor extended frontally towards the frontal language region of “Broca” as well. In P3, the tumor was located in the frontal and insular gyrus, in proximity or possibly with minimal involvement of the inferior frontal gyrus, that is, “Broca,” but not the posterior temporoparietal regions. Tumor locations in all patients were in the vicinity of the arcuate fasciculus (AF).

### 2.2. Procedure: Operation, Neuroimaging, and Pathological Findings

Between July 2011 and June 2013, patients were treated with awake brain surgery given the tumor location in or near presumed critical language regions. Electrical stimulation was carried out at cortical and subcortical level with a bipolar electrode. Object naming and repetition tasks were administered during stimulation, whereas more extensive language testing was conducted using the Dutch Linguistic Intraoperative Protocol (DuLIP) with spontaneous speech monitoring during resection [16].

Localization of the tumor was determined by a neuroradiologist using 3D T1-weighted images and 2D T2-weighted images. The pre- and postoperative tumor volume was calculated by manual delineation of 3-dimensional deviant signal intensity on T2-weighted MR images using Osirix version 4.1.2. (http://www.osirix-viewer.com/). Postoperative MRI scans were assessed at 6 months after surgery. The extent of the resection was calculated as the fraction (%) of the difference between the preoperative and postoperative volume divided by the preoperative volume. The histological type of the tumor (astrocytoma, oligodendroglioma, and oligoastrocytoma) and the World Health Organization (WHO) grade (2007) were determined by a neuropathologist, from tissue obtained during the tumor resection.

### 2.3. Neuropsychological Assessment

Pre- and postoperatively, we administered an extensive neuropsychological test-protocol (Table 2).* Language* tests are as follows: Boston Naming Test (BNT) or object naming (DuLIP), action naming, category and letter fluency, and Aachen Aphasia Test (AAT) subtests: repetition, writing to dictation, reading aloud, and Token Test.* Memory* tests are as follows: 15-word test (imprinting, recall); digit span.* Attentional and executive functions* tests are as follows: design fluency, Trail Making Tests A and B, and Stroop Color Word Tests I–III. Based on the normative data, *z*-scores were computed to compare performance of the patients to healthy controls. A clinical impairment is reflected by a *z*-score between −1.5 and −2; a pathological impairment is reflected by a *z*-score of ≥−2. Postoperatively, P1 and P2 were tested at 6 weeks and 6 months and P3 and P4 at 3 months and 1 year. The study was approved by the Ethical Committee of Erasmus MC Rotterdam and University of Brussels. All patients gave written informed consent.

## 3. Results

Tumor volume ranged from 1.46 cm^3^ to 108 cm^3^. Pathological examination of tumor tissue obtained during resection revealed a LGG (WHO grade II) in P1 and P3 and a HGG in P2 (WHO grade IV) and in P4 (WHO grade III). The extent of resection (EoR) ranged from 58 to 89%. P1, P2, and P4 underwent postoperative radiotherapy (33 fraction doses of 1.8 Gy). All patients used anticonvulsants pre- and postoperatively (see Table 2).

### 3.1. Neuropsychological Assessment: Pre-, Intra-, and Postoperative Course (See Table 3)

#### 3.1.1. P1: Low-Grade Glioma in “Wernicke's” Area and Near AF

Preoperatively, the cognitive functions of P1 were intact (*z* ≥ −1.5). During operation, speech arrest occurred after stimulation at the precentral gyrus (primary motor cortex) at the level of the mouth. Postoperatively, at 6 weeks a clinical deficit in a memory test was observed (15 WT imprinting; *z* = −1.60) which recovered at 6 months. No other cognitive deficits were observed (*z* ≥ −1.5).

#### 3.1.2. P2: High-Grade Glioma in “Wernicke's” Area Near AF

Preoperatively, P2 had clinically or pathologically significant impairments in language (letter fluency; *z* = −1.90, AAT Token Test; *z* = −5.35), and attention and executive deficits (TMT B: *z* = −1.60, TMT BA: *z* = −1.90, Stroop I: *z* = −2.00, Stroop II: *z* = −2.20, and Stroop III: *z* = −1.90). During surgery cortical stimulation in the posterior superior temporal gyrus/angular gyrus triggered speech arrest. During stimulation of the AF, phonemic paraphasia occurred; these increased during resection near the AF, at which point resection was terminated. At 6 weeks postoperatively new deficits were found in language (object naming: *z* = −6.28, action naming: *z* = −2.94, and category fluency: *z* = −2.50), in memory (15 WT imprinting: *z* = −3.90, 15 WT recall: *z* = −3.10, and digit span: *z* = −3.00), and in attention and executive functions (design fluency: productivity: *z* = −1.88, TMT A: *z* = −4.10). There was an increase of the preoperative deficits in language (letter fluency: *z* = −2.70, AAT Token Test: *z* = −10.81) and in attention and executive functions (TMT B: *z* = −4.30, TMT BA: *z* = −2.70, Stroop I: *z* = −5.40, Stroop II: *z* = −4.40, and Stroop III: *z* = −3.40). At 6 months postoperatively, improvement was observed in 1 subtest within the language domain (action naming: *z* = −1.37) and in the attention and executive functions (TMT A: *z* = −1.50), but further deterioration was found in another subtest (design fluency: productivity: *z* = −2.05).

#### 3.1.3. P3: Low-Grade Glioma in “Broca's” Area and Near AF

**Figure 3:**
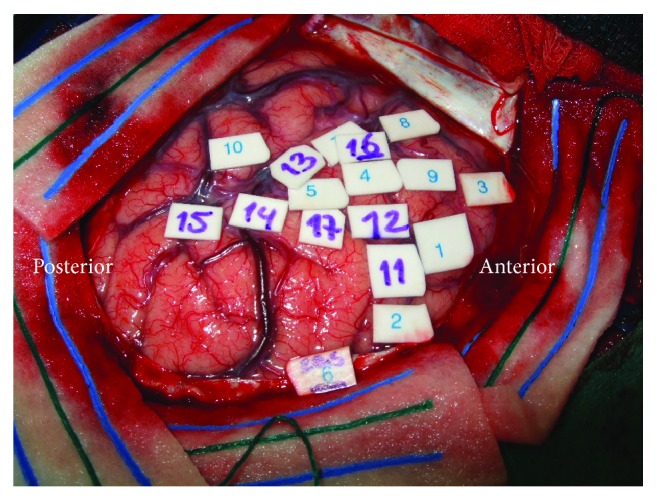
Intraoperative mapping P3. Cortical positive sites**:** speech arrest (1, 5, 7–9, and 13-14), dysarthria (2–4), neologism (10, 15), phonemic paraphasia (10, 15), and contraction of tongue (11-12, 16-17).

**Figure 4:**
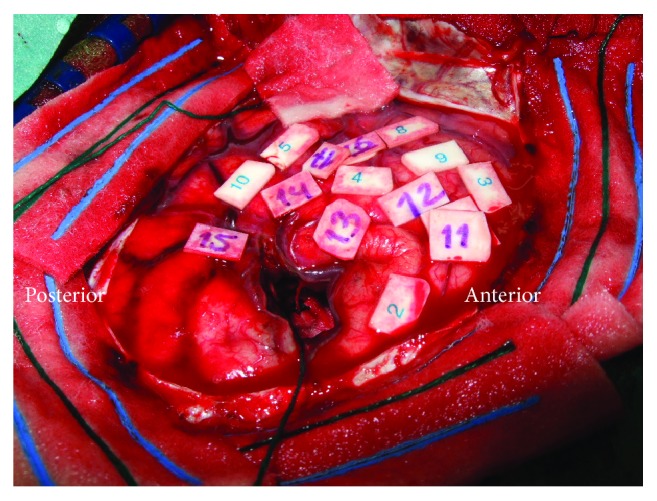
Resection cavity P3.

Preoperatively P3 was clinically impaired in memory (15 WT recall: *z* = −1.60) and had selective pathological impairments in executive functioning (TMT B: *z* = −2.40, TMT BA: *z* = −3.00). During surgery speech arrest occurred with stimulation of the inferior frontal gyrus, below the motor cortex, and the parietal lobe (see Figure 3). Phonemic paraphasia and neologisms were elicited at the temporoparietal junction. At the subcortical level near the AF also phonemic paraphasia was elicited. At the motor cortex, stimulation triggered dysarthria and contraction of the tongue. At the end of resection, perseverations occurred at which point resection was terminated (see Figure 4). Postoperatively at 3 months, the patient had recovered from the observed preoperative impairments in memory (15 WT recall: *z* = −0.50) and executive functioning (TMT B: *z* = 0.30, TMT BA: *z* = −0.40), which remained stable during the follow-up of 1 year (15 WT: *z* = 1.00, TMT B: *z* = −0.20, and TMT BA: *z* = −0.90). No other impairments were present.

#### 3.1.4. P4: High-Grade Glioma in “Wernicke's” Area with Extension to “Broca's” Area and Near AF

Preoperatively, P4 had no cognitive disorders. During surgery speech arrest was found when the inferior frontal gyrus was stimulated, below the motor cortex and in the temporal lobe. Phonemic paraphasia was elicited in the parietal lobe. Resection was terminated when perseverations occurred. At 3 months postoperatively, P4 developed a deficit in language (AAT repetition: *z* = −4.17) which had recovered at 1 year (*z* = −1.39) and in attention and executive functions (TMT BA: *z* = −1.60) which also recovered at 1 year (*z* = −0.60).

## 4. Discussion

A detailed examination of cognitive functions was conducted in 4 patients with brain tumors in or near “classical” language areas Broca, Wernicke, and the AF before and after awake surgery. Given the tumor localization it is remarkable that only in one patient (P2) a language disorder was present preoperatively. Our study revealed mixed cognitive profiles at pre- and postoperative time-points. Two patients (P1 and P4) showed relatively intact cognitive performance, which may be explained by neural plasticity (i.e., reorganization of functions). By contrast, the other two cases (P2 and P3) demonstrated impairments in several cognitive domains pre- and/or postoperatively. These different findings are in line with a “dynamic” or “hodotopical” brain as opposed to a “static” or “topological” viewpoint [6]. Several factors may be related to the plastic potential of the brain such as different corticosubcortical networks (localization), tumor grade (low versus high), tumor volume, EoR, and the use of anticonvulsants and/or adjuvant therapy with irradiation.

P1 with a tumor in Wernicke's area appeared to have intact cognition, apart from a temporary clinical memory deficit at 6 weeks postoperatively. Intact cognition after glioma surgery in Wernicke's area has been reported previously [21]. By contrast, a multicognitive disturbed profile at pre- and postoperative level was found in P2 who had a very similar tumor localization. This implies that the “classical” language area Wernicke can also be related to other cognitive functions or that cognitive functions are disturbed when language is impaired (partly) in line with the model proposed by Coello et al. [22]. However, it is not possible to make strong assumptions about the interdependency of deficits in different cognitive domains. In P2, a simultaneous decline of language, memory, and executive functions at T2 illustrates this phenomenon where both verbal and nonverbal tasks deteriorated (verbal fluency and design fluency). Surprisingly, P3 with a tumor near Broca's area did not suffer from preoperative language deficits probably due to functional reorganization. Instead, impairments in the domains of memory and executive functions were observed which recovered within 3 months. P4 with a large tumor extending to Broca's and Wernicke's area had generally intact language performance, again in contrast with the “classical” language model, apart from a temporary decline on a repetition task at 3 months after surgery. Recently, the sensitivity of a repetition task was demonstrated in the intraoperative stimulation setting especially in or near the AF [23]. In all our patients the tumor was also located in or near the AF. Surgery in this area can cause a decline in phonological language performance [24]. A variety of pre- and postoperative cognitive disturbances in our patients demonstrate that this subcortical tract (AF) is not only associated with phonology. This has previously been observed in patients with lesions with a different etiology [25]. Hence, preservation of AF during surgery appears to be mandatory for the surveillance of (further) cognitive decline. Despite the detection of intraoperative language positive sites in all patients, different postoperative cognitive outcomes were observed. Tumor resection in proximity of a language positive site, but also preoperative language deficits, can be a risk factor for postoperative aphasia [26].

Apart from localization and the intraoperative procedure, the differential pre- and postoperative cognitive profiles in our patients could be attributed to tumor related factors, such as tumor grade. Noll et al. [27] found that patients with grade IV gliomas present with poorer preoperative cognitive performance (verbal learning, processing speed, executive functioning, and language) than patients with lower-grade gliomas (II, III). These differences were not related to tumor size, seizure status, and anticonvulsants or steroid use which points to evidence of a so-called “lesion momentum”: faster growing tumors may be associated with more severe cognitive impairments. Our results are partly consistent with this line of reasoning. P2 with a high-grade glioma showed a preoperative disturbed cognitive profile (deficits in language, memory, and attention/executive functioning). Preserved cognitive functions in P1 were possibly facilitated by the slow growth rate of a low-grade tumor allowing “typical” functional reorganization (i.e., 4 mm p/y), that is, preoperative cognitive plasticity. A faster growth rate of a high-grade tumor, as in P2, could have more aggressively affected these preoperative cognitive functions. However, results in P3 and P4 do not concur with this hypothesis: P3 with a LGG presented disturbances of memory, attention, and executive functions whereas P4 with a HGG demonstrated overall intact cognitive performance. It is possible in this case (P4) that, due to fast tumor growth, mainly suppression of functional areas occurs, whereas the integrity of white matter bundles associated with function remains intact. Herbet et al. [28] showed via a probabilistic atlas that reorganization at subcortical level, in proximity to white matter tracts, could be less optimal than at cortical level. In addition, Trinh et al. [29] demonstrated that a subcortical injury was an independent predictor for longer-term neurological impairments underlining the importance of preservation of subcortical tracts. It may also be possible that genetic tumor mutation is associated with cognition: IDH1-mutant wild-type (isocitrate dehydrogenase), more aggressive than IDH1-mutant tumors, appeared to be associated with more severe cognitive impairments possibly hindering neuroplasticity [30].

Another intervening factor influencing preoperative cognitive performance could be tumor volume. Habets et al. [13] found that larger brain tumors in the left hemisphere were associated with poorer executive functioning. This explanation may hold for P2 who has a relatively large preoperative tumor volume and is suffering from more serious cognitive deficits compared to P1 with a smaller tumor and intact cognition. However, P3 and P4 showed the reversed pattern, with P4 having a larger tumor with intact cognitive performance and P3 with a smaller tumor and deficits.

EoR may have played a role as P2 and P4 underwent a more extensive tumor resection than P1 and P3. However, a recent follow-up study did not reveal a relation between EoR and cognitive decline [5]. Currently, there is only evidence that a more extensive resection is associated with longer survival in both LGG and HGG patients [31]. In addition, in all patients resection was conducted according to individual subcortical functional boundaries.

In general, (stimulation-induced) seizures and the use of anticonvulsants can be a risk factor for deficits in cognitive performance [32]. Deficits in information processing, attention, and executive functions were found to be related to the use of anticonvulsants in long-term glioma survivors (at least 1 year after diagnosis) in the absence of seizures [33]. In our patients, the use of medication may have added to cognitive defects or postoperative decline. However, all patients took anticonvulsants both before and after surgery, which makes it hard to draw any firm conclusions. Apart from antiepileptic drugs, radiotherapy may also have had negative effects on cognitive performance [34]. P1, P2, and P4 were treated with radiotherapy, of whom P2 and P4, but not P1, showed postoperative cognitive deterioration during the administration of irradiation. In addition, in all patients a “safe” fraction dose of maximally 2 Gy per session was administered, which is known to be associated with relative stable cognition for several years after irradiation [35].

Finally, some other factors should be taken into account when interpreting our results. Handedness may have interfered with the results, as P1 was left-handed as opposed to P2– P4. Language organization in left-handed people is not always consistent and can be represented in a more widespread network than in right-handed people [36]. However, all patients had tumors in the language dominant hemisphere as attested with fMRI. The detection of crossed cerebellar activation may add to the identification of language lateralization in (left-handed) brain tumor patients [37]. From these 4 cases, it is clear that cognitive functions cannot be related to a certain location in the brain. Unfortunately, we do not know when and until which period improvement of specific cognitive functions exactly takes place. Follow-up measurements were not administered at similar time-points in all patients, namely, 6 weeks or 3 months for early and 6 months or 1 year for late follow-up. However, a recent outcome study found that the postoperative interval of 3 and 6 months is crucial for language improvement, whereas recovery of the executive functions appeared to take longer than 6 months [38]. Evidently, larger subgroups with patients with a comparable brain tumor localization and, for instance, tumor grade need to be analyzed to investigate the different courses that underlie functional neural plasticity. No postoperative fMRI and diffusion tensor imaging studies were available; therefore, it is difficult to account for reorganization at both the structural and functional level.

Patients with brain tumors in “classical” language areas do not necessarily present language (or other cognitive) disturbances. Surgery can either improve or deteriorate (existing) cognitive (impairments) functions. The findings of these case studies provide therefore further support for neural plasticity within a “hodotopical” framework. It remains uncertain to which extent and which factors, such as localization, tumor grade, volume, EoR, and/or adjuvant therapy, contribute to neural plasticity. Hence, an extensive examination of cognitive functions with larger (sub)groups taking into account localization, tumor, and treatment related factors will elucidate prognostic factors of the plastic potential of the brain.

## Figures and Tables

**Table 1:** Demographic and clinical characteristics. Y = years, Hem. Dom. = hemispheric dominance, M = male, L = left, R = right, AF = arcuate fasciculus, O = oligodendroglioma, GBM = glioblastoma, AO = anaplastic oligodendroglioma, EoR = extent of resection, and AED = antiepileptic drugs.

Case	Gender	Age (y)	Education (y)	Handedness	Onset symptoms	Hem. Dom.	Cortical area	Subcortical area	WHO grade (2007)	Histology	EoR (%)	AED	Adjuvant therapy
P1	M	45	15	L	Seizure	L	Temporoparietal (“Wernicke”)	AF	II (low)	O	58.34	Yes	Radio
P2	M	63	12	R	Language problems	L	Temporoparietal (“Wernicke”)	AF	IV (high)	GBM	88.68	Yes	Radio
P3	M	41	16	R	Seizure	L	Frontal and insular (“Broca”)	AF	II (low)	O	52.73	Yes	No
P4	M	33	15	R	Seizure	L	Temporoparietal and frontal (“Wernicke” and “Broca”)	AF	III (high)	AO	81.48	Yes	Radio

**Table 2:** Neuropsychological assessment.

	Cognitive abilities and description of task
*Language tests*	
AAT [17]	
Repetition	Repeating phonemes, words, and sentences
Writing to dictation	Writing words and sentences on dictation
Reading out loud	Reading aloud words and sentences
Token Test	Comprehension of, pointing to, and manipulating geometric forms
Boston Naming Test [18]	Naming 60 pictures, presented in order of word frequency and word difficulty
Category fluency	Flexibility of verbal semantic thought: categories (e.g., animals) (within 1 min)
Letter fluency	Flexibility of verbal phonological thought: letters D, A, and T (within 1 min)
DuLIP [16]	
Syntactic fluency	Flexibility of verbal grammatical thought, producing verbs (within 1 min)
Object naming^1^	Word finding: naming objects
Action naming	Word finding, grammar: naming actions
*Memory tests*	
Digit span for/backward [19]	Verbal learning of digits: repeating the list of digits forward/backward
15-word test [19]	Verbal learning of words
Learning	Immediate recall: learning a list of 15 words, immediate recall for 5 times
Recall	Delayed recall: learning a list of 15 words, 1 delayed recall
Recognition	Delayed recognition: 1 delayed recognition out of 30 words
*Attentional & executive tests*	
Trail Making Test (TMT) [19]	
Trail Making Test A	Visuomotor speed, attention: connecting numbers in ascending order
Trail Making Test B	Divided attention/mental flexibility: connecting alternating numbers and letters
Stroop Color Word Test [19]	
Stroop I	Mental speed, selective attention: reading color words
Stroop II	Mental speed, selective attention: naming colors
Stroop III	Mental speed, selective attention: naming colors of printed words denoting another color
Design fluency [20]	Nonverbal fluency, attention, motor speed, visuoperceptual and constructional abilities

^1^Flanders: object naming from DuLIP, the Netherlands: Boston Naming Test.

**(a):** 

P1			
	Preoperative results	Postoperative results (6 w)	Postoperative results (6 m)
*Language*			
Object naming (DuLIP)	0.13	0.63	0.63
Action naming (DuLIP)	0.76	0.76	0.76
Category fluency	0.10	−1.00	0.45
Letter fluency	1.10	−0.10	0.50
AAT Token Test	0.83	0.83	0.83
*Memory*			
15 WT imprinting	−0.10	*−1.60* ^*∗*^	0.10
15 WT recall	−0.60	−0.60	−0.20
Digit span	0.67	0.33	1.00
*Attention/executive functions*			
Design fluency productivity	2.05	1.41	2.33
Design fluency flexibility	1.28	0.58	0.67
Design fluency strategy	1.08	2.05	1.28
TMTA	1.10	1.20	1.60
TMTB	1.40	1.70	2.10
TMTBA	0.90	1.20	1.40
Stroop I	−0.30	−0.40	−0.20
Stroop II	1.50	1.20	1.80
Stroop III	1.60	2.40	1.90
Stroop interference	1.00	2.30	1.20

**(b):** 

P2			
	Preoperative results	Postoperative results (6 w)	Postoperative results (6 m)
*Language*			
Object naming (DuLIP)	−0.64	**−6.28** ^*∗∗*^	**−4.40** ^*∗∗*^
Action naming (DuLIP)	−0.80	**−2.94** ^*∗∗*^	−1.37
Category fluency	−1.20	**−2.50** ^*∗∗*^	**−2.30** ^*∗∗*^
Letter fluency	*−1.90* ^*∗*^	**−2.70** ^*∗∗*^	**−2.40** ^*∗∗*^
AAT Token Test	**−5.35** ^*∗∗*^	**−10.81** ^*∗∗*^	**−5.35** ^*∗∗*^
*Memory*			
15 WT imprinting	−0.64	**−3.90** ^*∗∗*^	**−3.50** ^*∗∗*^
15 WT recall	−0.80	**−3.10** ^*∗∗*^	**−2.70** ^*∗∗*^
Digit span	−1.20	**−3.00** ^*∗∗*^	**−3.00** ^*∗∗*^
*Attention/executive functions*			
Design fluency productivity	0.13	*−1.88* ^*∗*^	**−2.05** ^*∗∗*^
Design fluency flexibility	−0.25	−1.13	0.00
Design fluency strategy	0.84	−0.47	0.25
TMTA	0.20	**−4.10** ^*∗∗*^	*−1.50* ^*∗*^
TMTB	*−1.60* ^*∗*^	**−4.30** ^*∗∗*^	**−3.40** ^*∗∗*^
TMTBA	*−1.90* ^*∗*^	**−2.70** ^*∗∗*^	**−3.10** ^*∗∗*^
Stroop I	**−2.00** ^*∗∗*^	**−5.40** ^*∗∗*^	**−5.00** ^*∗∗*^
Stroop II	**−2.20** ^*∗∗*^	**−4.40** ^*∗∗*^	**−5.10** ^*∗∗*^
Stroop III	*−1.90* ^*∗*^	**−3.40** ^*∗∗*^	**−3.40** ^*∗∗*^
Stroop interference	−0.60	−1.20	−0.30

**(c):** 

P3			
	Preoperative results	Postoperative results (3 m)	Postoperative results (1 y)
*Language*			
Boston Naming Test	−0.60	−0.27	−1.27
Category fluency	1.10	1.57	−0.46
Letter fluency	0.00	0.54	1.08
AAT Token Test	−0.10	−0.83	−0.83
AAT repetition	1.39	0.83	0.83
AAT reading aloud	0.54	0.54	0.54
AAT writing to dictation	0.54	0.54	0.54
*Memory*			
15 WT imprinting	−1.40	−0.40	0.40
15 WT recall	*−1.60* ^*∗*^	−0.50	1.00
*Attention/executive functions*			
TMTA	0.50	1.10	1.00
TMTB	**−2.40** ^*∗∗*^	0.30	−0.20
TMTBA	**−3.00** ^*∗∗*^	−0.40	−0.90
Stroop I	0.60	0.60	1.10
Stroop II	−0.30	−0.30	0.70
Stroop III	0.70	0.10	1.10
Stroop interference	1.10	0.30	0.80

**(d):** 

P4			
	Preoperative results	Postoperative results (3 m)	Postoperative results (1 y)
*Language*			
Boston Naming Test	−0.21	−0.74	0.05
Category fluency	0.75	−1.16	0.73
Letter fluency	−1.08	−1.26	−1.44
AAT Token Test	−0.47	0.99	−0.10
AAT repetition	0.28	**−4.17** ^*∗∗*^	−1.39
AAT reading aloud	−0.49	−0.49	0.54
AAT writing to dictation	0.27	−0.27	0.00
*Memory*			
15 WT imprinting	−1.30	1.50	−0.10
15 WT recall	−0.70	0.00	0.40
*Attention/executive functions*			
TMTA	0.70	0.00	0.90
TMTB	−0.60	−1.40	0.00
TMTBA	−1.10	*−1.60* ^**∗**^	−0.60
Stroop I	0.80	−0.90	−0.30
Stroop II	1.10	0.00	−0.50
Stroop III	1.20	−0.50	−0.30
Stroop interference	0.60	−0.70	0.00
